# Remodelling of Nucleus-Vacuole Junctions During Metabolic and Proteostatic Stress

**DOI:** 10.1177/25152564211016608

**Published:** 2021-05-27

**Authors:** Verena Kohler, Sabrina Büttner

**Affiliations:** 1 Department of Molecular Biosciences, The Wenner-Gren Institute, Stockholm University, Stockholm, Sweden; 2 Institute of Molecular Biosciences, University of Graz, Graz, Austria

**Keywords:** glucose, metabolism, NVJ, Snd3, stress response, quiescence

## Abstract

Cellular adaptation to stress and metabolic cues requires a coordinated response of different intracellular compartments, separated by semipermeable membranes. One way to facilitate interorganellar communication is via membrane contact sites, physical bridges between opposing organellar membranes formed by an array of tethering machineries. These contact sites are highly dynamic and establish an interconnected organellar network able to quickly respond to external and internal stress by changing size, abundance and molecular architecture. Here, we discuss recent work on nucleus-vacuole junctions, connecting yeast vacuoles with the nucleus. Appearing as small, single foci in mitotic cells, these contacts expand into one enlarged patch upon nutrient exhaustion and entry into quiescence or can be shaped into multiple large foci essential to sustain viability upon proteostatic stress at the nuclear envelope. We highlight the remarkable plasticity and rapid remodelling of these contact sites upon metabolic or proteostatic stress and their emerging importance for cellular fitness.

## Introduction

Adaptation to changing environments is key to maintaining cellular and organismal survival. For microbial cells, evolutionary success depends on their ability to sense and rapidly adapt to fluctuating availability of nutrients or exposure to stress. Cellular metabolism needs to be adjusted to allow growth and division in times of plenty and to sustain homeostasis and stress response when nutrients are limiting (Zhang and Cao, 2017). Optimal resource allocation requires sensing and signalling of extracellular conditions and a subsequent metabolic rewiring and cellular remodelling to adjust organellar functions. Direct physical contact between distinct organelles at membrane contact sites is thought to play a key role in the intracellular relay of biochemical information (Gatta and Levine, 2017). These sites serve as metabolic hubs and rapidly change size and abundance in response to various types of external and internal cues, thereby optimizing and fine-tuning organellar function to respective cellular needs. Contact sites allow the transfer of information in form of ions, lipids or small metabolites between distinct cellular subcompartments, thus providing a connection between spatially separated biochemical reactions within each organellar microenvironment (Xu and Huang, 2020). In cooperation with classical signalling pathways and vesicular transport, this form of interorganellar communication links the metabolic activities in different organelles to guarantee overall cellular homeostasis. Bridging of different organellar membranes at contact sites is established by specialized tethering machineries that shape the different contacts via protein-protein or protein-lipid interaction and serve to recruit and position additional proteins to tailor contact function or enforce contact formation (Eisenberg-Bord et al., 2016; Scorrano et al., 2019). Sites of contact have been reported across the eukaryotic kingdom and between virtually all organelles, with functions ranging from lipid flux and organelle fission to the involvement in degradative pathways like autophagy (Eisenberg-Bord et al., 2016; Levine and Patel, 2016; Gatta and Levine, 2017; Kohler et al., 2020). While being able to rigidly tether two opposing membranes, contact sites have also been shown to dynamically change in size, abundance and molecular composition in response to a variety of external and internal cues. Despite extensive research on the dynamic aspects of contact sites (Eisenberg-Bord et al., 2016; Nettebrock and Bohnert, 2019; Scorrano et al., 2019; Bohnert, 2020; Prinz et al., 2020), the signalling events triggering this remodelling are far from being understood. Reshaping of contact sites often occurs simultaneously at several organellar contact zones and includes the relocalization of distinct proteins from one site to another, supporting the notion that these contacts form a coordinated network able to collectively respond to specific stimuli (Hönscher et al., 2014; Elbaz-Alon et al., 2015; Bean et al., 2018; Hariri et al., 2018; Bohnert, 2019; Prinz et al., 2020).

## The Making and Shaping of Nucleus-Vacuole Junctions

The budding yeast *Saccharomyces cerevisiae* represents a prime model organism for studies on the molecular architecture of contact sites and their regulation and multifaceted functions. Several processes and proteins associated with different contacts have been first described in yeast, followed by the identification of the mammalian counterparts (Elbaz-Alon et al., 2014; Hönscher et al., 2014; Hönscher and Ungermann, 2014; Eden, 2016; Malia and Ungermann, 2016; Wong et al., 2018). The first contact sites discovered in yeast are the nucleus-vacuole junctions (NVJs), connecting the vacuole with the perinuclear endoplasmic reticulum (ER) and thus the nuclear envelope (Pan et al., 2000). A few years ago, a potential equivalent in mammalian cells was described, tethering the ER to endosomes (Hönscher and Ungermann, 2014; Eden, 2016). Some NVJ-associated proteins find their counterparts in this mammalian membrane contact site and distinct functions are indeed conserved, in particular lipid transfer. Still, contacts between the ER and the endolysosomal system in higher eukaryotes are more complex than yeast NVJs, both in respect to structure and function. The extent of organellar contact as well as the tethering proteins that compose the contact site seem highly dependent on the state of endosomal maturation, and functions of these contact sites range from positioning, movement and fission of endosomes to the modulation of receptor signalling (Di Mattia et al., 2020).

The size and appearance of NVJs can range from small foci to large patches, establishing highly dynamic zones of organellar proximity between the ER as the key compartment for lipid synthesis and the vacuole as cellular waste bin, recycling facility and storage depot. Accumulating evidence places NVJs at the centre stage of lipid metabolism: these junctions recruit an array of proteins involved in different aspects of lipid metabolism and transport, and serve as a platform for lipid droplet (LD) biogenesis under stress (Pan et al., 2000; Jeong et al., 2017; Hariri et al., 2018).

**Figure 1:**
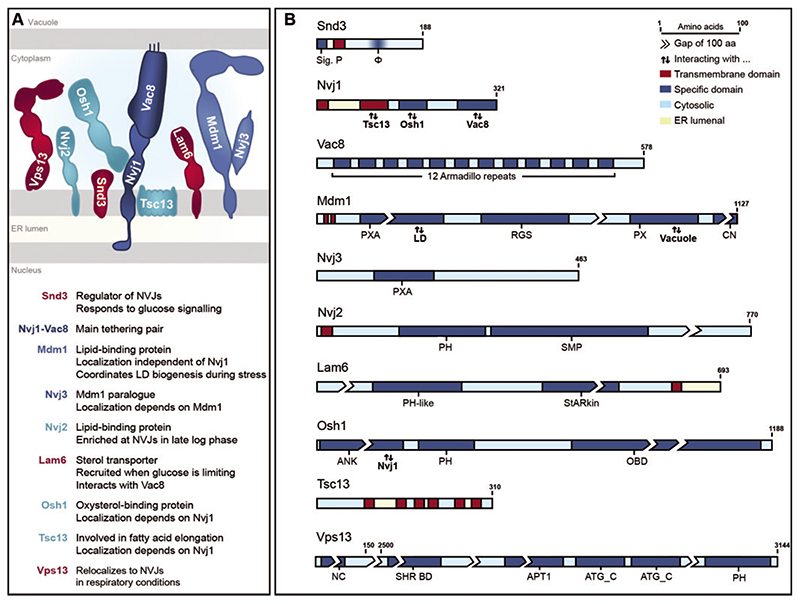
Overview of nucleus-vacuole junctions. A: The main tethering pair of NVJs (dark blue), alternative tethers (mid blue), accessory proteins involved in lipid metabolism (light blue) and regulatory proteins (red) are depicted and their main function is given. Please note that orientations and domain borders are represented according to predictions, but relative protein size and structure are not true to scale. B: Schematic representation of main NVJ components. Protein sizes are true to scale with breaks of 100 amino acids, where necessary. Transmembrane domains (red) and specific domains (dark blue) are highlighted. Protein regions facing the cytosol are shaded in light blue, those towards the ER lumen are highlighted in yellow. Sig.P. = signal peptide; *ϕ* = hydrophobic region; PXA = Phox-associated; LD = lipid droplet; RGS = regulator of G protein signalling; PX = Phox; CN = C-terminal nexin; PH = pleckstrin homology; SMP = synaptogamin-like-mitochondrial-lipid binding protein; StARkin = domain closely resembling StART (steroidogenic acute regulatory protein-related lipid-transfer) domain; ANK = ankyrin; OBD = oxysterol-binding domain; NC = N–terminal chorein domain; SHR BD = SHORT_ROOT transcription factor binding domain; APT1 = aberrant pollen transmission 1 protein; ATG_C = Autophagy-related protein C-terminal domain. Information regarding domain borders were taken from respective literature, Uniprot and SGD (Kohlwein et al., 2001; Toulmay and Prinz 2012; Henne et al., 2015; Murley et al., 2015; Jeong et al., 2017; Manik et al., 2017; Rzepnikowska et al., 2017a, 2017b; Dziurdzik et al., 2018; Tosal-Castano et al., 2021).

The interaction between the principle NVJ tethering pair, the perinuclear ER-resident protein Nvj1 and the palmitoylated protein Vac8 on the vacuolar membrane, represents a prerequisite for NVJ formation (Figure 1) (Pan et al., 2000). Nvj1 anchors its N-terminal domain to the inner nuclear membrane to span the ER lumen (Millen et al., 2008), and interaction with the Armadillodomain protein Vac8 is facilitated via its C-terminus (Jeong et al., 2017). These tethers allow the recruitment of additional contact components, mostly proteins involved in lipid metabolism. This includes the oxysterol-binding protein Osh1, associating with cytoplasmic parts of Nvj1 via its ankyrin domain, and the enoyl-CoA reductase Tsc13, an essential component of the fatty acid elongation machinery localized in the ER membrane, which contacts Nvj1 via its transmembrane domain (Kohlwein et al., 2001; Levine and Munro, 2001; Kvam and Goldfarb, 2004; Kvam et al., 2005; Jeong et al., 2017; Manik et al., 2017). Nvj2, one of seven SMP-proteins in yeast, all of which localize to membrane contact sites, is a lipid-binding protein with a role in ceramide metabolism (Liu et al., 2017; Tamura et al., 2019). Nvj2 was found to prominently enrich at NVJs in late-logarithmic growth but seems to partly dissociate when cells enter stationary phase (Toulmay and Prinz, 2012). The integral ER-protein Mdm1 contacts the vacuole at the junctions via its lipid-binding PX-domain, thereby acting as an additional tether, and promotes LD biogenesis via localized fatty acid activation (Hariri et al., 2016, 2018). Interestingly, Mdm1 as well as its paralogue Nvj3 are targeted to the junctions independently of the main tether protein Nvj1 to establish organellar proximity (Henne et al., 2015), while for instance Osh1 and Tsc13 are recruited to the NVJs via Nvj1. The sterol-transfer protein Lam6 requires Vac8 to localize to the junctions, where it mediates the formation of sterol-enriched lipid microdomains on the vacuolar membrane upon glucose limitation and facilitates contact expansion (Elbaz-Alon et al., 2015; Murley et al., 2015). Vps13, a peripheral membrane protein that is enriched at the contacts between ER and vacuole under respiratory conditions, is involved in phospholipid transport (Lang et al., 2015; Park et al., 2016; Bean et al., 2018). Both yeast and mammalian Vps13 localize to and coordinate the size of several other contact sites, most likely due to altered lipid distribution, and only transiently associate with NVJs and ER-endosomal contacts, respectively (Lang et al., 2015; Park et al., 2016; Rzepnikowska et al., 2017a, 2017b; Bean et al., 2018; Gao and Yang, 2018). Though orthologues of several NVJ-associated proteins involved in lipid metabolism, such as Vps13, Mdm1, Nvj2, and Osh1, are present in higher eukaryotes, counterparts of the main tethering pair Nvj1-Vac8 have been only identified in other fungi (Millen et al., 2008; Beh et al., 2012; Toulmay and Prinz, 2012; Helle et al., 2013; Manik et al., 2017; Rzepnikowska et al., 2017b; Garapati and Mishra, 2018; Singh et al., 2020). Thus, especially membrane contact site-situated effector proteins involved in lipid metabolism seem to be conserved over species boundaries, as elaborated in several excellent reviews (Eden, 2016; Henne, 2017; Scorrano et al., 2019; Bohnert, 2020; Di Mattia et al., 2020), while principle structural tethers seem to vary substantially.

While these additional NVJ residents regulate NVJ dynamics, plasticity and function, and are enriched at these contact zones under specific conditions, they are not required for contact formation *per se*. In our recent work, we identified the integral ER protein Snd3 as a novel component of NVJs (Figure 1) and show that this protein is crucial to establish NVJs (Tosal-Castano et al., 2021). We demonstrate that Snd3 interacts with and stabilizes Nvj1 once it is inserted into the ER membrane. In absence of Snd3, Nvj1 is rapidly removed from the ER via ER-associated degradation (ERAD). As the topology of Nvj1 is rather unique, spanning both membranes of the nuclear envelope (Millen et al., 2008), stabilization of this protein by Snd3 is required to preclude premature retro-translocation and proteasomal degradation via ERAD (Tosal-Castano et al., 2021). After successful insertion of Nvj1 into the nuclear envelope, primordial NVJs are formed via interaction between the main tethering pair Vac8 and Nvj1, followed by recruitment of other NVJ-associated proteins (Kohlwein et al., 2001; Levine and Munro, 2001; Kvam and Goldfarb, 2004; Kvam et al., 2005). Upon glucose exhaustion or during respiratory growth on glycerol, Snd3 itself is targeted to the NVJs, facilitating contact site expansion (Tosal-Castano et al., 2021). While more and more proteins are being identified to decorate the NVJs, a comprehensive analysis of the NVJ proteome under different metabolic regimes is still lacking.

## NVJ Expansion Upon Glucose Exhaustion and Entry Into Quiescence

**Figure 2:**
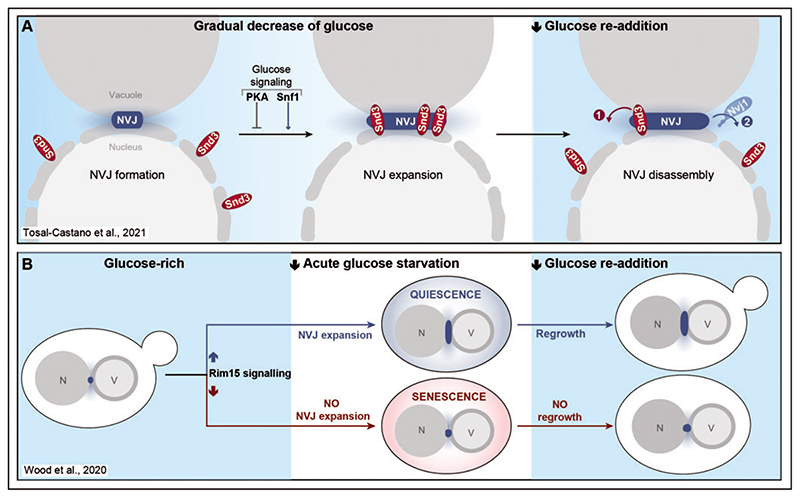
NVJ expansion upon glucose depletion and entry into quiescence. A: The ER-protein Snd3 both enriches at and governs the expansion of the nucleus-vacuole junctions (NVJs) when yeast cells grow into glucose exhaustion, a process regulated by central glucose signalling pathways. Upon glucose replenishment, Snd3 quickly redistributes along the perinuclear ER, followed by a slower disassembly of NVJs. B: Expansion of NVJs is a robust predictor of cell fate, when yeast cells acutely starved for glucose face quiescence versus senescence decisions. In a clonal population, cells that efficiently expand the NVJs upon acute starvation and cell cycle arrest are more likely to enter quiescence, defined as the ability to re-enter the cell cycle when glucose is replenished again. Vice versa, cells becoming senescent are less likely to show this contact site expansion during acute glucose removal. For details, please see main text. PKA = protein kinase A; N = nucleus; V = vacuole.

Upon entry into stationary phase or in response to stress, NVJ size increases, and the small NVJ foci visible at the interface between the nucleus and the vacuole grow into large, expanded patches (Pan et al., 2000; Kohlwein et al., 2001; Levine and Munro, 2001; Hariri et al., 2018). Along this line, we could show that glucose exhaustion triggers NVJ expansion and recruitment of Snd3 as additional contact site resident (Figure 2A). When cells rapidly divide in high glucose, Snd3 is evenly distributed throughout the ER but already essentially contributes to the formation of primordial NVJs via stabilisation of the main tether Nvj1 once it is translocated. As soon as glucose becomes limiting, Snd3 is targeted to the junctions, which coincides with the enrichment of Nvj1 and massive NVJ expansion. *Vice versa*, glucose re-addition to glucose-starved cells triggers the rapid dissociation of Snd3 from the junctions, followed by the slow disassembly of these contacts (Tosal-Castano et al., 2021). We further show that recruitment of Snd3 to the junctions and concomitant NVJ expansion is controlled by central glucose signalling pathways, establishing a link between metabolic rewiring and contact site dynamics (Figure 2A). The protein kinase A (PKA) as well as the AMP-dependent kinase Snf1 represent two evolutionary conserved branches of the metabolic signalling network, activated in response to high glucose or glucose depletion, respectively (Hedbacker and Carlson, 2008; Zhang and Cao, 2017; Stasyk and Stasyk, 2019). Mimicking the presence of glucose via (i) genetic inactivation of the inhibitory subunit of PKA, resulting in hyperactive PKA signalling, or (ii) disruption of AMP-dependent Snf1 signalling, prevented Snd3 targeting to and Nvj1 enrichment at the contact sites despite glucose depletion (Tosal-Castano et al., 2021).

In response to glucose exhaustion, cells exit the cell cycle and enter quiescence, a non-proliferative state with rather heterogenous properties associated with high stress resistance and longevity. Entry into quiescence has been shown to be accompanied by a drastic remodelling of distinct organelles and cellular structures (Sagot and Laporte, 2019). Recent work from *Wood et al*. demonstrates that this organellar remodelling entails a prominent expansion of NVJs that serves as predictor of cell fate (Wood et al., 2020). Using abrupt restriction of glucose to force exit from cell cycle, the authors assessed biomarkers for nutrient signalling, interorganellar cross-talk, stress response and organelle homeostasis to predict quiescence *versus* senescence decisions of individual cells within an isogenic yeast population (Figure 2B) (Wood et al., 2020). In an elegant approach using multi-colour time-lapse imaging and automated single-cell analysis, they monitored protein-fluorophore chimeras, including Nvj1, throughout growth in high glucose, acute starvation for several hours and subsequent glucose replenishment. To maintain cellular viability upon abrupt starvation, cells need to quickly adapt by rewiring cellular homeostasis and exit cell cycle, which requires central metabolic signalling networks, including an inhibition of PKA signalling and the activation of the AMPK/Snf1 pathway (Zhang and Cao, 2017). Interestingly, *Wood et al*. discovered that a high abundance of the nutrient-signalling protein kinase Rim15 (Swinnen et al., 2006) prior to acute starvation is a strong indicator for successful entry into quiescence and thus the ability to regrow when glucose is replenished. In addition, Nvj1 as a marker for interorganellar connectivity turned out to be a strong predictor of cell fate. Cells within a clonal population that rapidly expanded NVJs during acute starvation were more likely to become quiescent, while senescent cells mostly lacked NVJ expansion. Still, the presence of Nvj1 was not required for entry into quiescence (Wood et al., 2020). Thus, even though NVJs seem to respond to signalling pathways coordinating the cellular path towards quiescence or senescence upon acute starvation, a distinct physiological role for Nvj1 in this process has not been identified yet. While indeed NVJ expansion might merely represent a phenotypic marker of quiescence, it is also feasible that for instance Mdm1, which was recently established as an alternative NVJ tether (Henne et al., 2015; Hariri et al., 2018), compensates for the absence of Nvj1, allowing the formation of organellar contact despite lack of the main tether.

## NVJ Remodelling to Resolve Proteostatic Stress at the Nuclear Envelope

**Figure 3:**
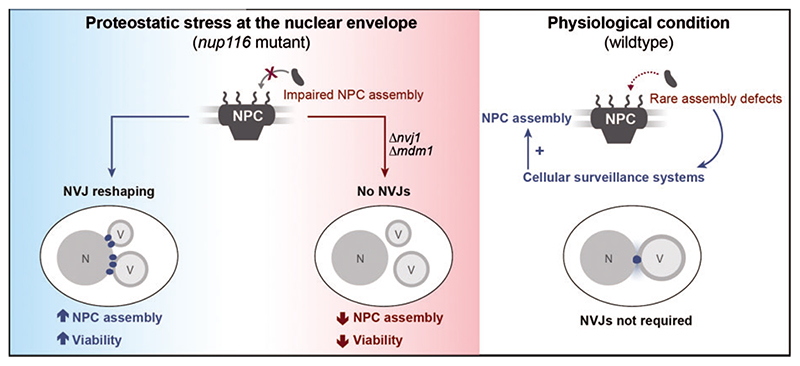
NVJ expansion upon defective NPC assembly is required to sustain cellular fitness. Proteotoxic stress upon impaired nuclear pore complex (NPC) assembly in a *nup116* mutant triggers NVJ reshaping, leading to increased contact formation and multiple, large NVJ foci. This alleviates NPC assembly perturbations and promotes viability. Survival upon genetic disruption of NPC assembly (*nup116* mutant) requires NVJs, while under physiological conditions (wildtype) other cellular surveillance systems are in place to resolve the rare NPC assembly defects, rendering NVJs unnecessary for cellular viability. For details, please see main text. N = nucleus; V = vacuole.

Expansion of the contact region between the nucleus and the vacuole is not limited to nutritional stress but also occurs upon exposure to other stresses, in particular ER stress (Hariri et al., 2018). Establishing physical contact between the ER and the vacuole as main catabolic compartment, the NVJs are in a prime position to contribute to quality control of the nuclear envelope. The degradation of damaged material of the perinuclear ER and nucleolar proteins at the NVJs via piecemeal microautophagy of the nucleus (PMN), a selective form of autophagic recycling, represents one of the best-characterized functions of these contact sites (Millen et al., 2009; Golam Mostofa et al., 2018; Otto and Thumm, 2021). Here, parts of the nuclear envelope invaginate into the vacuolar lumen and small vesicles are pinched off to be digested. In addition to a core set of autophagy-related (Atg) proteins required for final stages of PMN (Krick et al., 2008), also the lipid microenvironment at NVJs is suggested to contribute to PMN. Pinching off these bleblike structures necessitates high membrane curvature, which is facilitated by incorporation of very-long chain fatty acids into phospholipids. Accordingly, insufficient fatty acid elongation compromises biogenesis of PMN vesicles (Kvam et al., 2005). Beyond this established role of NVJs during PMN and thus bulk removal of parts of the nuclear envelope, NVJs seems to be involved in another, more specific branch of protein quality control of the nucleus. *Lord and Wente* recently reported that the presence of NVJs was crucial to remove misassembled nuclear pore complexes (NPCs). Using mutants deficient in the assembly of NPCs, multi-subunit complexes composed of several nucleoporins that facilitate nucleocyto-plasmic transport, the authors could show that NVJs are critical to sustain cellular fitness upon perturbation of NPC assembly (Figure 3) (Lord and Wente, 2020). In contrast to metazoans, yeast undergoes a closed mitosis, thus NPCs have to be inserted into the intact nuclear envelope during interphase in a complex process (Boettcher and Barral, 2013). As NPC assembly defects are observed in replicatively aged cells (Rempel et al., 2019), it might be interesting to test for a possible contribution of NVJs to the quality control of the nuclear envelope in old mother cells.

*Lord and Wente* demonstrated that a mutant form of Nup116, a nucleoporin suggested to promote NPC biogenesis by stabilizing assembly intermediates (Onischenko et al., 2017), is defective in NPC assembly at elevated temperatures, resulting in clustered nucleo-porin patches that are degraded in an autophagy-dependent manner to remodel the nuclear envelope (Lord and Wente, 2020). Proteostatic stress upon disturbed NPC assembly triggered a prominent reshaping of NVJs: while the regions of contact between the nuclear envelope and the vacuole increased, Nvj1 did not appear in a single, enlarged patch but rather in multiple foci, decorating contact sites with numerous small vacuoles. Interestingly, Snd3 (alias Pho88), which the authors used as a marker for the perinuclear ER, did not concentrate at these Nvj1-decorated foci upon perturbed NPC assembly. Thus, while remodelling and expansion of the contacts occurs upon nutritional and proteostatic stress, the molecular composition of these enlarged NVJs seems to differ.

Mdm1, which coordinates LD biogenesis at NVJs upon nutrient limitation (Hariri et al., 2018), decorated the multiple NVJs just as Nvj1 upon NPC misassembly. Increased Mdm1 recruitment coincided with a clustering of LDs around the contact zones. Interestingly, these remodelled NVJs cooperate with LDs to relieve proteo-static stress upon NPC misassembly and to maintain viability, and loss of either one compromised cellular fitness. As inactivation of LD biogenesis or NVJ formation did not affect the viability of unperturbed wildtype cells (Lord and Wente, 2020), other surveillance systems might be in place to deal with occasional disturbances of NPC assembly in physiological conditions (Thaller et al., 2019). In this line, NPCs and nucleoporins were shown to be degraded via selective autophagy in unperturbed wildtype cells without the need for NVJ assembly or functional PMN (Tomioka et al., 2020). However, upon mutation or additional stress that hamper NPC assembly, possibly overloading available proteostatic capacity under resting conditions, NVJs emerge as important factors to relieve nuclear envelope stress. As fully assembled NPCs are usually excluded from the nuclear envelope region establishing the NVJs (Severs et al., 1976; Pan et al., 2000; Roberts et al., 2003), direct PMN-mediated degradation seems rather unlikely. Still, impaired NPCs and in particular assembly intermediates could be direct cargo of PMN at NVJs. Alternatively, NVJs might serve an indirect role in the removal of misassembled NPCs, for instance via spatial organization of LDs, which contribute to the generation of specific lipid microenvironments and have been shown to assist in the removal of misfolded proteins to relief proteostatic stress.

## Spatial Organisation of LDs at NVJs: A Contingency Route Upon Excessive Proteotoxic Stress?

Upon metabolic stress, NVJs serve as platforms for the organisation and biosynthesis of a specific LD subpopulation (Hariri et al., 2018). LDs bud off from the ER network and consist of a core of neutral lipids surrounded by a monolayer of phospholipids. These highly dynamic and versatile organelles function as lipid reservoirs to meet the cell’s requirements in times of nutrient limitation and to support the detoxification of excess fatty acids or harmful lipid species. Moreover, accumulating evidence points towards a crucial role of LDs in the removal of misfolded proteins from the cytosol and especially the ER (Jason et al., 2015; Moldavski et al., 2015; Henne et al., 2018). Under conditions of severe proteotoxic stress, LDs are assumed to serve as ‘protein sink’ to support the proteasomal system in aggregate clearance. Accordingly, cells devoid of LDs remove misfolded proteins less efficiently (Moldavski et al., 2015). ER stress induced by acute lipid imbalance causes a massive increase in LD number, in particular at sites of ER aggregates (Jason et al., 2015). Those LDs were found to be loaded with polyubiquitinated proteins and the ER chaperone Kar2, suggesting that LDs indeed sequester damaged proteins at these sites. Subsequent vacuolar degradation of these LDs and their proteotoxic cargo was facilitated by a selective form of autophagy termed microlipophagy, in which the vacuole directly engulfs its cargo (Jason et al., 2015).

Thus, the LD population decorating the periphery of the ER-vacuole contact zone might be in a prime position to sequester misfolded proteins from the ER and facilitate efficient microphagic removal due to the close proximity to the vacuole. Accordingly, not only LD biogenesis and NVJ formation but also Atg1 as key autophagy regulator were required for degradation of NPC assembly intermediates (Lord and Wente, 2020). One might speculate that NVJ-mediated spatial organisation of LDs aids in localized protein removal and subsequent vacuolar engulfment, supporting the proteasome in the turnover of misfolded proteins from the ER upon persisting stress. Mdm1, which facilitates the biosynthesis and clustering of LDs at the periphery of ER-vacuole contacts upon limitation, was also found enriched at the junctions upon compromised NPC assembly. Hence, Mdm1 might link NVJs to LD organisation when cells are subjected to metabolic and proteostatic stress (Henne et al., 2015; Hariri et al., 2018). The notion that LDs serve as ‘escape hatches’ for the retrieval of misfolded proteins is well-supported experimentally (Ploegh, 2007; Jason et al., 2015; Moldavski et al., 2015). However, whether specifically the LDs decorating the NVJs upon proteotoxic stress serve a dedicated function in protein quality control of the ER remains to be shown.

## Outlook

Accumulating studies on the dynamic remodelling and physiological relevance of NVJs highlight these contact sites as hubs to integrate metabolic cues and stress responses to determine cellular fate (Hariri et al., 2018; Lord and Wente, 2020; Wood et al., 2020; Tosal-Castano et al., 2021). While compromised NVJ formation remains without major effect on cellular viability in unperturbed cells (Wood et al., 2020), disruption of specific aspects of proteostasis, as shown for deficiencies in NPC assembly (Lord and Wente, 2020), necessitate efficient NVJ formation for survival.

This might be the case for other scenarios of cumulative cellular damage, for instance during cellular ageing (Chen et al., 2020, 2021; Li et al., 2020; Moreno and Aldea, 2020; Mouton et al., 2020). Though NVJs are not essential to drive entry into quiescence upon glucose starvation, prominent NVJ expansion seems to accompany this transition (Wood et al., 2020). Cells can persist in a quiescent state for a long time, awaiting re-entry into cell cycle upon more favourable conditions. Thus, NVJs might serve to spatially concentrate specific enzymatic activities necessary to maintain cellular homeostasis when cells have to persist for an extended period of time in a dormant state characterized by slow metabolism. In support of this, NVJs act as platforms for the local synthesis of a distinct LD subpopulation when cells face nutritional stress (Hariri et al., 2018, 2019). On the other hand, NVJ expansion prior to entry into quiescence might support immediate restart of cell growth and proliferation once nutrients are available again. This could provide an evolutionary advantage when microbial cells have to rapidly increase protein and lipid biogenesis to populate their environment and propagate their heritage (Sun and Gresham, 2021). It is tempting to speculate that NVJs represent some kind of storage units for a distinct set of proteins, in particular for enzymes involved in lipid metabolism, keeping them available for rapid re-distribution as soon as cells encounter conditions suitable for growth. In this line, Snd3 is released from enlarged NVJ patches in starved cells within minutes after glucose is replenished (Tosal-Castano et al., 2021). Whether Snd3 might for instance be involved in the interplay between NVJs and LDs, which is suggested to mitigate proteostatic stress in the nuclear envelope to sustain viability (Lord and Wente, 2020), remains to be shown. As a specific relocalization of Snd3 to the NVJ foci in these conditions was not observed, this protein might respond selectively to nutritional cues. Still, lack of Snd3 as crucial factor for NVJ expansion reduces competitive fitness in large-scale studies (Giaever et al., 2002; Qian et al., 2012), suggesting important functions of this protein to sustain survival of a clonal population.

While global responses to general nutrient deprivation are in place to regulate entrance into quiescence, cells also possess genetic programs specifically tailored to starvation of distinct macronutrients (Boer et al., 2010; Conway et al., 2012; Sun et al., 2020). Not only the set of genes necessary to maintain viability during starvation but also the concomitant cellular remodelling differs depending on the depleted nutrient (Klosinska et al., 2011; Sagot and Laporte, 2019). Thus, NVJs might differentially contribute to entry into or exit from quiescence depending on the respective nutrient scarcity. The protein levels of Nvj1 as the main tether are upregulated in glucose-starved and stationary cells (Pan et al., 2000; Tosal-Castano et al., 2021), most probably controlled by the stress-response element in the promoter region of Nvj1 (Moskvina et al., 1998). Still, the proteome of expanded NVJs is not known yet and will most likely differ depending on the specific cellular status. For instance, proteostatic stress restricted to the nuclear envelope or ER stress drive reshaping and expansion of the NVJs, but a general induction of protein misfolding via heat shock does not affect NVJ appearance (Hariri et al., 2018; Lord and Wente, 2020).

In sum, NVJs emerge as hubs that integrate distinct cellular stress signals and metabolic cues to regulate cell fate. The molecular machineries driving NVJ remodelling and tailoring NVJ molecular composition to the respective cellular needs are far from being understood and represent an exciting challenge for further research.
